# Malaria in Zhejiang Province, China, from 2005 to 2014

**DOI:** 10.4269/ajtmh.15-0080

**Published:** 2015-08-05

**Authors:** Hualiang Chen, Linong Yao, Lingling Zhang, Xuan Zhang, Qiaoyi Lu, Kegen Yu, Wei Ruan

**Affiliations:** Zhejiang Provincial Center for Disease Control and Prevention, Hangzhou, China

## Abstract

To summarize the changing epidemiological characteristics of malaria in Zhejiang Province, China, we collected data on malaria from the Chinese Notifiable Disease Reporting System (NDRS) and analyzed them. A total of 2,738 malaria cases were identified in Zhejiang Province from 2005 to 2014, of which 2,018 were male and 720 were female. Notably, only 7% of malaria cases were indigenous and the other cases were all imported. The number of malaria cases increased from 2005 to 2007, peaked in 2007, and then decreased from 2007 to 2011. There were no indigenous cases from 2012 to 2014. Of all cases, 68% of cases contracted *Plasmodium vivax*, 27% of cases contracted *P. falciparum*, and two cases contracted *P. malariae*. About 88% of malaria cases during 2005–2011 occurred yearly between May and October, but the number of malaria cases in different months during 2012–2014 was similar. The median age was 33 years, and 1,892 cases occurred in persons aged 20–50 years. The proportion of businessmen increased and the proportion of migrant laborers decreased in recent years. The median time from illness onset to confirmation of malaria cases was 5 days and it decreased from 2005 to 2014. Some epidemiological characteristics of malaria have changed, and businessmen are the emphases to surveillance in every month.

## Introduction

Despite widespread elimination and control efforts during the 20th century, malaria continues to be the most important parasitic disease known to humankind. According to the World Health Organization (WHO) estimate in 2014, there were 3.2 billion people at risk of being infected with malaria and developing disease; 198 million cases of malaria and 584,000 deaths occurred globally in 2013.^1^ Over the span of the last century, almost half of the world's countries have successfully eliminated malaria.^2^ Although great success has been achieved since the launch of national malaria control program in 1955, malaria remains a serious public health problem in China.^3^–^5^ The Chinese Notifiable Disease Reporting System (NDRS), which was initiated in the 1950s, is the fundamental communicable disease surveillance system in China. After the outbreak of severe acute respiratory syndrome (SARS) in 2003, the Chinese government strengthened the construction of public health information system. On January 1, 2004, the Real-Time Notifiable Infectious Disease Reporting System was put into use nationwide, realizing the timely online monitoring of individual cases, which marks a leap in the surveillance of communicable diseases in China.

Zhejiang Province is located in southeastern China, adjacent to Anhui Province, where malaria is endemic. Malaria incidence decreased in recent years in Zhejiang Province, but there were hundreds of imported malaria cases every year and epidemiological characteristics changed.^6^,^7^ This stimulated us to analyze the updated epidemiological characteristics of malaria cases in recent years.

## Methods

### Case definition.

Indigenous malaria was defined as any case infected within the province where it was diagnosed; in contrast, imported malaria was defined as a malaria case whose origin could be traced to an area of transmission outside the province where the diagnosis of malaria was made.^8^,^9^

### Data collection.

Daily disease surveillance data on malaria from 2005 to 2014 were obtained from the NDRS. Information of cases included gender, age, occupation, residential address, type of disease, date of onset, and date of confirmation.

### Data analysis.

Data were analyzed using the Statistical Package for the Social Sciences (SPSS v19; SPSS, Chicago, IL). Categorical variables were summarized by frequencies and numerical variables were summarized by means with standard deviations if normally distributed and medians, interquartile ranges (IQRs), and ranges if not normally distributed.

### Ethical approval.

Experimental research reported in this study has been performed with the approval of the ethics committee of Zhejiang Provincial Center for Disease Control and Prevention (Zhejiang CDC). Human research was carried out in compliance with the Helsinki Declaration. All participants provide their written informed consent to participate in this study.

## Results

**Figure 1.**
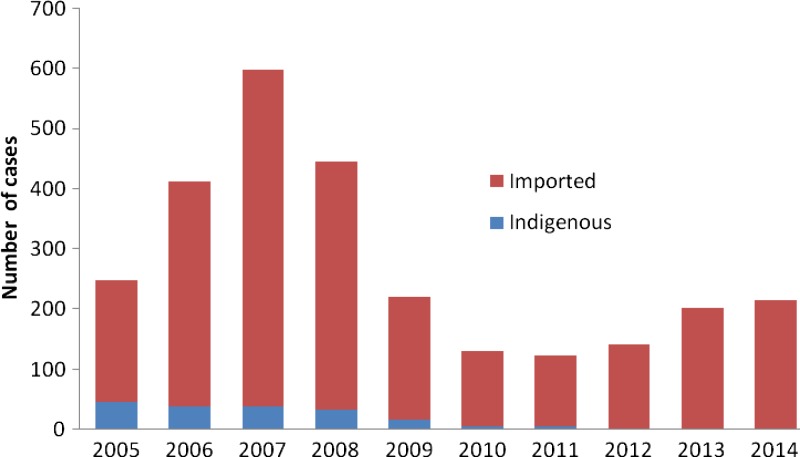
Annual distribution of malaria cases in Zhejiang Province from 2005 to 2014.

A total of 2,738 malaria cases were identified in Zhejiang Province from 2005 to 2014, of which 2,018 were male and 720 were female. Of note, only 7% (183/2,738) of malaria cases were indigenous and the other cases were all imported. As shown in Figure 1

, the number of malaria cases increased from 2005 to 2007 and peaked in 2007, and then the number of malaria cases decreased from 2007 to 2011. Although the number of malaria cases increased from 2012 to 2014, there were no indigenous cases in these 3 years (Figure 1).

Of all cases, 68% (1,865/2,738) of reported cases contracted *Plasmodium vivax*, 27% (731/2,738) of reported cases contracted *P. falciparum*, and two cases contracted *P. malariae*, which were imported from Nigeria and Libya, respectively (Table 1). Because of the lack of diagnostic test in some counties before 2009, 140 cases were unclassified.

**Figure 2.**
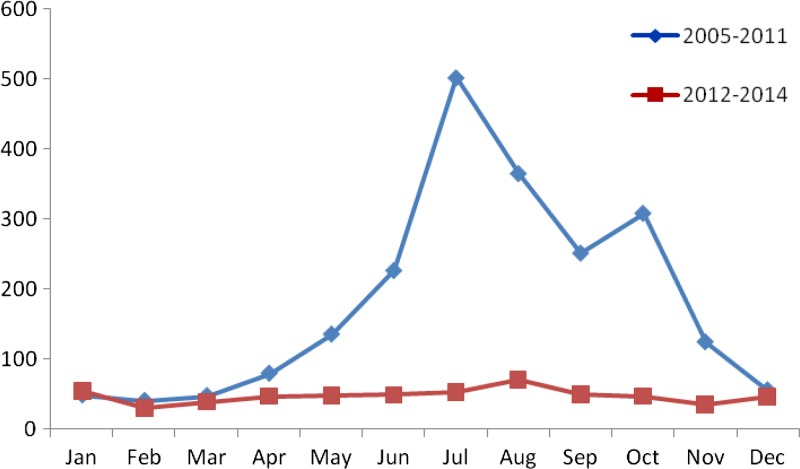
Monthly distribution of malaria in Zhejiang Province from 2005 to 2014.

Malaria cases were reported in every month; the majority (88%) of malaria cases during 2005–2011 occurred yearly between May and October (Figure 2

). However, the number of malaria cases of different months during 2012–2014 was similar.

**Figure 3.**
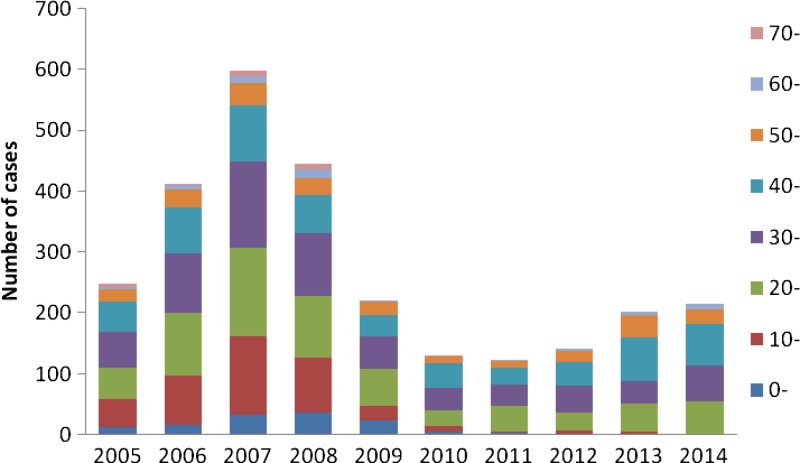
Age distribution of malaria in Zhejiang Province from 2005 to 2014.

**Figure 4.**
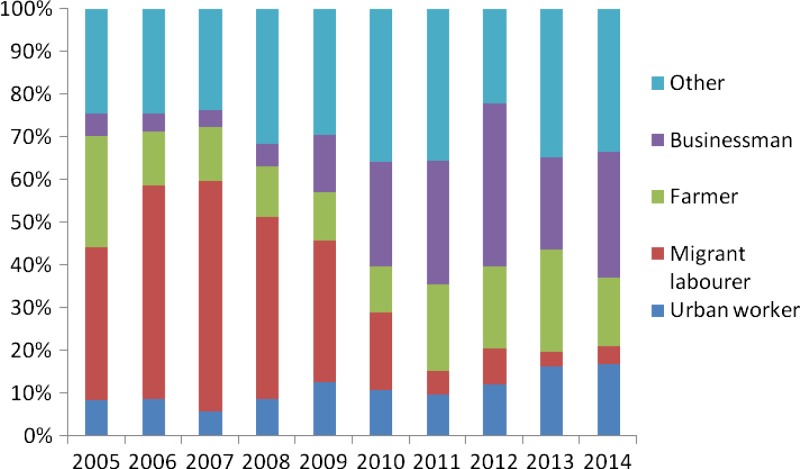
Occupation distribution of malaria in Zhejiang Province from 2005 to 2014.

The median age of reported malaria cases was 33 (range: 1–84 years) and 1,892 (69%) cases occurred in persons aged 20–50 years (Figure 3

). Migrant laborers, farmers, businessmen, and urban workers constituted 72% (1,965/2,738) of malaria cases (Figure 4

). Moreover, the proportion of businessmen increased and the proportion of migrant laborers decreased in recent years.

**Figure 5.**
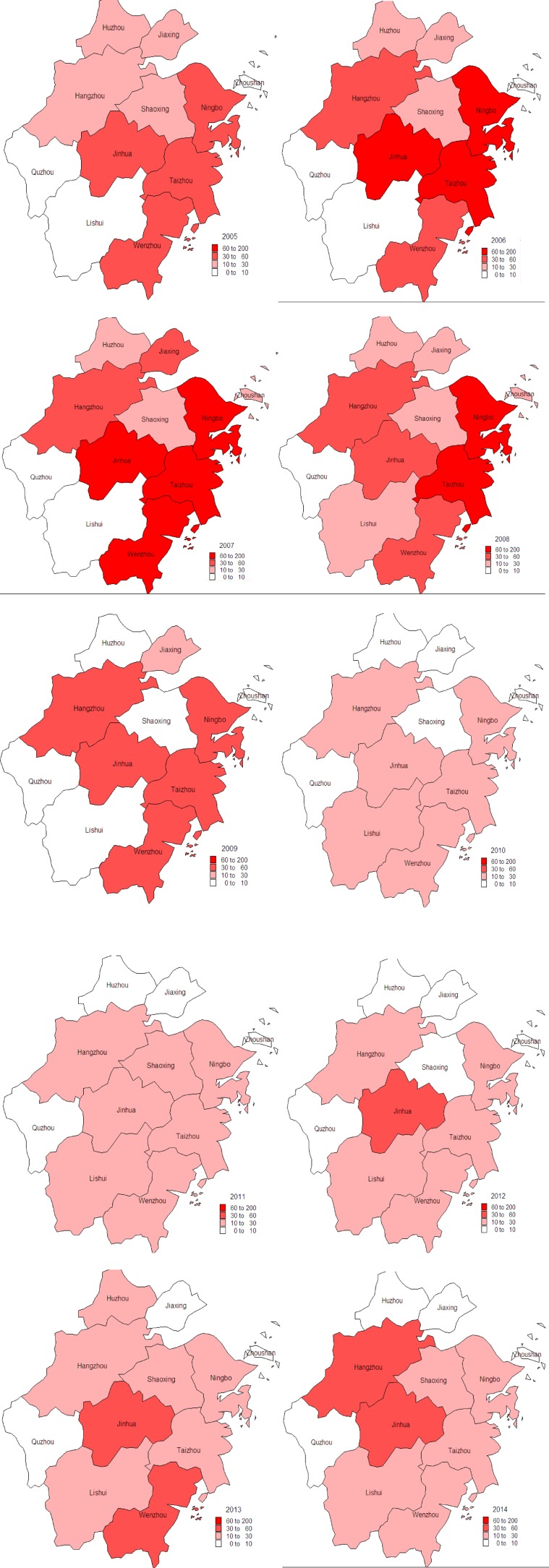
Geographical distribution of malaria cases in Zhejiang Province from 2005 to 2014.

Malaria cases were reported in all 11 cities of Zhejiang Province from 2005 to 2014. However, malaria cases from Ningbo, Jinhua, Taizhou, Wenzhou, and Hangzhou accounted for 79% of cases, and these five cities reported 582, 494, 425, 345, and 308 malaria cases, respectively (Figure 5

).

From 2005 to 2009, no information about the origins of imported cases was investigated, but these detailed data were collected from 2010 to 2014. Among 808 imported cases, 626 cases were from Africa, 117 from Asia, 59 from Chinese provinces, and only six cases from countries of other continents (Table 2). Nigeria, Ghana, Equatorial Guinea, Angola, Congo, and Cameron were the dominant origins of imported cases. Anhui Province where malaria was prevalent was the main origin of imported cases within China.

The median time from illness onset to confirmation of all cases was 5 days (IQRs: 3–8 days; range: 0–332 days). In addition, the median time from illness onset to confirmation decreased from 2005 to 2014 as shown in Table 3.

## Discussion

Although the elimination of malaria has helped to shrink the global malaria map, this has unveiled a more complex pattern of malaria epidemiology globally.^2^ Imported cases of malaria into the nonendemic regions are being increasingly recognized as a new public health challenge.^10^ In our study, the majority of malaria cases from 2005 to 2011 were imported and all cases from 2012 to 2014 were imported indicating that the control of imported cases was of vital importance for the elimination of malaria in Zhejiang Province.

To eliminate malaria in Zhejiang Province, plan on elimination of malaria was made by Provincial Health Department, Provincial Development and Reform Commission, Provincial Education Department, Provincial Science and Technology Department, Provincial Economic and Information Commission, Provincial Public Security Department, Provincial Finance Department, Provincial Commerce Department, Provincial Entry–Exit Inspection and Quarantine Bureau, Provincial Radio, Film and Television (TV) Bureau, and Provincial Tourist Administration in 2010. According to the plan, many departments collaborated with each other to eliminate malaria in Zhejiang Province and comprehensive measures were conducted. First, the control and management of infection source were enhanced. Screening of malaria among patients with high fever was conducted in province, city, county, and town levels medical institutions. Cases were reported, treated, and managed soon after the confirmation. Second, control and prevention of mosquito were enhanced. The patriotic public health campaign was carried and breeding places of mosquito were removed. Moreover, residual spray was used to reduce density of mosquito and bed nets were used to prevent mosquito bites. Third, health education was enhanced through newspapers, TV, radio, and Internet. We also collaborate with Entry–Exit Department and Travel Bureau to educate methods or prevent malaria among individuals who went to or came from malaria-endemic areas. Finally, prevention and control of malaria among immigrants was enhanced. Entry-exit departments was in charge of screening of malaria cases among fever patients and the information on patients were reported to medical departments. Public security departments helped to trace malaria patients among immigrants. Nevertheless, mosquito density was high in malaria areas and businessmen were inevitably bit by mosquito. As a result, decades of malaria cases were imported in Zhejiang Province. This informed that elimination of malaria in endemic areas would contribute to the reduction of imported cases in nonendemic areas.

In our study, all cases contracted *P. vivax* or *P. falciparum* except in two cases where *P. malariae* caused malaria. The reasons might be that the majority of cases were imported and most of the imported cases came from Africa. If the majority of the cases were domestic, there might be more vivax cases. Furthermore, misdiagnosis might exist because of poor diagnostic capacity of county-level centers for diseases control and prevention. We noted that the majority of cases were *P. vivax* infection, which was similar to studies from Qatar, Kuwait, Kingdom of Bahrain, and the United Arab Emirates.^11^–^14^ Although *P. vivax* malaria is commonly considered nonsevere and has been historically termed as “benign tertian malaria,” new published studies showed that cases of *P. vivax* infection often resulted in increased hospitalizations, severe disease, and death than previously expected.^14^–^17^ These informed us that *P. vivax* malaria was the greatest cause of malaria morbidity in Zhejiang Province.

The majority of malaria cases during 2005–2011 occurred yearly between May and October, but the number of malaria cases in different months during 2012–2014 was similar. The reasons for the temporal pattern of 2005–2011 may include higher mosquito density and more outdoor activities between May and October. Therefore, people had more chances to be bitten by mosquito, increasing the number of malaria cases. All malaria cases were imported from 2012 to 2014, the number of cases was mostly related to the frequency of population migration.

The age and gender distribution suggested the predominance of male cases aged 20–50 years in Zhejiang Province from 2005 to 2014. The reasons might include that a large number of nonimmune adults aged 20–50 years such as travelers and laborers moving from low-transmission to high-transmission areas and that men have a higher occupational risk if they work in mines, fields, or forests at peak biting times, or migrate to malaria-endemic areas for work.^18^ Of note, the proportion of businessmen increased and the proportion of migrant laborers decreased in recent years. This might be due to trade between malaria-endemic areas and Zhejiang Province was improved in recent years.

Although malaria cases were reported in all 11 cities of Zhejiang Province from 2005 to 2014, the majority of cases were reported in Ningbo, Jinhua, Taizhou, Wenzhou, and Hangzhou. This may be associated with environmental and high frequency of travel and trade in these cities. The growth of rice in these cites provided breeding places for *Anopheles sinensis*, which increased the transmission probability of malaria in these areas. Some areas of these five cities were infection focus of malaria in the past. Furthermore, the number of imported cases was relative to the frequency of travel and trade to malaria-endemic areas. The frequency of travel and trade of the five cities was significantly higher than that in other areas.

Early treatment of malaria with an appropriate antimalarial medication is the most important factor in limiting progression to severe or complicated disease, and delayed diagnosis predicted fatal outcome and severe course of falciparum malaria.^19^ In our study, the median time from illness onset to confirmation of all cases was 5 days. Fortunately, the median time from illness onset to confirmation decreased from 2005 to 2014. This may be related to health education to international traveler, good access to health care, and scaling-up diagnostic testing.

In summary, the number of malaria cases decreased in recent years, and no indigenous cases were reported in Zhejiang Province from 2012 to 2014. Some epidemiological characteristics of malaria changed in recent years, the risk of imported malaria was similar among different months, and the businessmen accounted for the highest proportion. These results informed that imported cases were the emphases for the control and prevention of malaria, and measures should be taken in all months in Zhejiang Province. Measures to better intercept imported cases should include health education and preventive medication among travelers to malaria-endemic areas, the screening of malaria among travelers with high fever, and collaborating with the neighboring countries. In addition, other comprehensive measures, such as early case detection and prompt treatment, residual spraying, usage of bed nets, environmental and antilarval management, and monitoring of drug resistance, should also be conducted to achieve the elimination of malaria in Zhejiang Province.

## Figures and Tables

**Table 1:** Type distribution of malaria in Zhejiang Province from 2005 to 2014

	*Plasmodium vivax*	*P. falciparum*	*P. malariae*	Unclassified	Total
2005	218	15	0	16	249
2006	341	27	0	44	412
2007	520	34	0	44	598
2008	381	35	0	29	445
2009	172	46	0	3	221
2010	67	60	0	4	131
2011	41	83	0	0	124
2012	46	95	0	0	141
2013	46	156	0	0	202
2014	33	180	2	0	215
Total	1,865	731	2	140	2,738

**Table 2:** The origins of imported cases from 2010 to 2014

		2010	2011	2012	2013	2014	Total
Africa	Nigeria	10	19	13	33	49	124
	Ghana	3	4	14	41	13	75
	Equatorial Guinea	2	12	23	22	19	78
	Angola	14	15	9	18	20	76
	Congo	2	10	5	12	15	44
	Cameron	2	6	1	11	23	43
	Guinea	1	5	2	3	4	15
	Uganda	4	2	2	3	2	13
	Gabon	1	0	4	4	7	16
	Liberia	2	0	2	4	9	17
	Tanzania	3	2	1	2	2	10
	Côte d'Ivoire	1	2	2	3	4	12
	Sierra Leone	0	0	4	1	0	5
	Other African countries	8	12	21	26	31	98
Asian	Burma	5	4	13	4	2	28
	Cambodia	2	12	7	4	0	25
	India	8	2	5	6	2	23
	Pakistan	2	5	1	4	3	15
	Indonesia	0	1	2	4	5	12
	Vietnam	2	0	1	0	3	6
	Other Asian countries	2	1	5	0	0	8
China	Anhui	36	3	1	1	0	41
	Yunnan	4	0	2	1	0	7
	Other Chinese provinces	5	5	0	1	0	11
Countries of other continents		4	0	0	0	2	6

**Table 3:** The median time from illness onset to confirmation malaria in Zhejiang Province from 2005 to 2014

	Median	IQR	Range
2005	6	4–11	0–104
2006	6	4–9	0–205
2007	6	3–8.25	0–152
2008	6	3–8	0–332
2009	5	3–9	0–153
2010	4	2–9	0–61
2011	4	2–9	0–271
2012	3	1–7	0–140
2013	3	1–5	0–71
2014	3	1–5	0–167

IQR = interquartile range.
